# Phenotype-considered kinematically aligned total knee arthroplasty for windswept-deformity-associated osteoarthritis: surgical strategy and clinical outcomes

**DOI:** 10.1186/s43019-024-00220-x

**Published:** 2024-04-02

**Authors:** Cheng-En Hsu, Meng-Hsueh Tsai, Hsin-Ting Wu, Jen-Ting Huang, Kui-Chou Huang

**Affiliations:** 1 Sports Recreation and Health Management Continuing Studies-Bachelor’s Degree Completion Program, Tunghai University, Taichung, 407 Taiwan; 2 Department of Orthopedics Surgery, Taichung Veterans General Hospital, 1650 Taiwan Boulevard Sect. 4, Taichung, 40705 Taiwan; 3 Department of Orthopedics Surgery, Asia University Hospital, 222 Fuxin Rd., Wufeng District, Taichung, 41354 Taiwan; 4 Department of Occupational Therapy, Asia University, 500 Lioufeng Rd., Wufeng, Taichung, 41354 Taiwan

**Keywords:** Windswept deformities, Knee arthroplasty, Scoliosis, Leg length discrepancy, Kinematically aligned total knee arthroplasty

## Abstract

**Background:**

Windswept deformity (WSD) in relation to advanced osteoarthritis (OA) presents a significant surgical challenge in total knee arthroplasty (TKA). The primary goal of this study is to investigate the Prevalance of WSD associated osteoarthritis who have undergone total knee arthroplasty. The secondary goal is to explore the causes of WSD and its association with spinal deformity or leg length discrepancy in these patients. Finally, we evaluate the surgical outcomes of phenotype-considered kinematically aligned TKA (KA-TKA) in treating patients with WSD.

**Methods:**

A review was conducted on data from 40 knees of 33 WSD patients who underwent phenotype-considered KA-TKA from August 2016 to December 2020. Patient demographics, associated diseases, preoperative and postoperative knee alignment angles, range of motion (ROM), Oxford Knee Score (OKS), and Knee Society Score (KSS) were collected and analyzed. Subgroup analysis for comparing the results between valgus and varus knees were also performed.

**Results:**

Within the studied cohort of WSD patients, a substantial 64% displayed concomitant coronal spinal imbalance and 21% evidenced leg length discrepancy. Postoperative improvements were notable in knee alignments, ROM, OKS, and KSS following the application of the phenotype-considered KA-TKA approach. There were significant differences in the knee alignment angles, including mHKA, LDFA, and MPTA, between the valgus and varus side of knees (*P* = 0.018). However, no statistically significant difference were observed in the functional scores, comprising ROM, OKS, and KSS, between valgus and varus knees.

**Conclusions:**

A high percentage of patients with WSD exhibited coronal spinal imbalance and leg length discrepancy. Phenotype-considered KA-TKA effectively provided alignment targets for the treatment of both varus and valgus knees in patients with WSD, achieving excellent short-term outcomes and acceptable knee alignment.

## Introduction

Windswept deformity (WSD) is a bilateral condition in which one knee exists in valgus deformity while the other shows varus deformity [1]. The causes of WSD include scoliosis, pelvic obliquity, osteoarthritis of hip, developmental dysplasia of hip, leg length discrepancy, and arthropathies from the knee joint [2, 3]. Total knee arthroplasty is the definite treatment for terminal knee arthropathy. WSD has high surgery complexity and requires individualized surgical strategies to achieve good radiographic and clinical results [4–7]. However, there is a paucity of literature discussing individualized surgical strategies for this deformity.

Traditionally, restoring the lower extremity malalignment to neutral alignment is the main purpose of TKA, which is known as the mechanically aligned TKA (MA-TKA) method. However, the kinematically aligned (KA) technique as an alternative to MA has aroused increasing interest in recent decades. KA-TKA has been reported to offer better patient satisfaction, functional outcome, faster recovery, soft tissue balance, and joint-line alignment than MA-TKA [8–13]. KA-TKA aims to find the predisease joint line and restore it [14]. Despite advancements, challenges persist in estimating an individual’s alignment prior to the onset of arthritis and determining an appropriate alignment target [9–12], To allow for more personalized alignment in KA-TKA, we previously studied the distribution of knee alignment among patients, leading to a categorization of the five most common phenotypes for alignment target setting. With the goal of establishing personalized alignment objectives for different knee types, we developed a phenotype-considered KA-TKA that adapts to the various knee phenotypes in the coronal plane [15, 16].

With a phenotype-considered approach to KA-TKA, individualized alignment goals can be set for different types of varus and valgus knees. This can aid in preoperative planning, particularly when setting alignment targets for varus and valgus knees. We hypothesized that this approach may be a good strategy to find an alignment target in patients with OA associated with WSD. This study aims to achieve three objectives: firstly, to establish the prevalence of WSD in patients with osteoarthritis who have undergone total knee arthroplasty; secondly, to investigate the incidence of associated spinal deformities or leg length discrepancies in patients with WSD; and thirdly, to explore the use of a personalized approach in treating varus and valgus knees separately, and to investigate the surgical outcomes of this phenotype-considered KA-TKA in the treatment of advanced OA knees in WSD patients.

## Patients and methods

### Study subjects

This study was designed as a retrospective analysis of data that were collected prospectively. The protocol of this study was approved by the institutional review board of a local medical center (IRB no. CMUH108-Rec1-088). From August 2016 to December 2020, data of 1250 patients who underwent TKA at a single institution by a single surgeon were reviewed. Standing full-length long-leg films were taken for all patients who underwent TKA, to evaluate limb alignment and leg length. Patients with WSD-related OA were included for analysis. The inclusion criteria were as follows: (1) mechanical hip–knee–ankle angle (mHKA) < −3° on the varus knee and mHKA > 3° on the valgus knee (valgus alignment being assigned a positive value) and (2) osteoarthritis with Kellgren–Lawrence grade III or IV. The excluding criteria were any forms of postoperative infection and follow-up time less than 1 year.

Thirty-three patients met the criteria and were included in the study. Seven of 33 (20%) patients received sequential total knee arthroplasty for the other leg. Forty total knee arthroplasties were performed in these 33 patients.

### Outcome evaluation

All radiographic images were digitally acquired and processed using a picture archiving and communication system (PACS) with a minimum measurement angle of 0.01° and length of 0.01 mm.

**Fig. 1:**
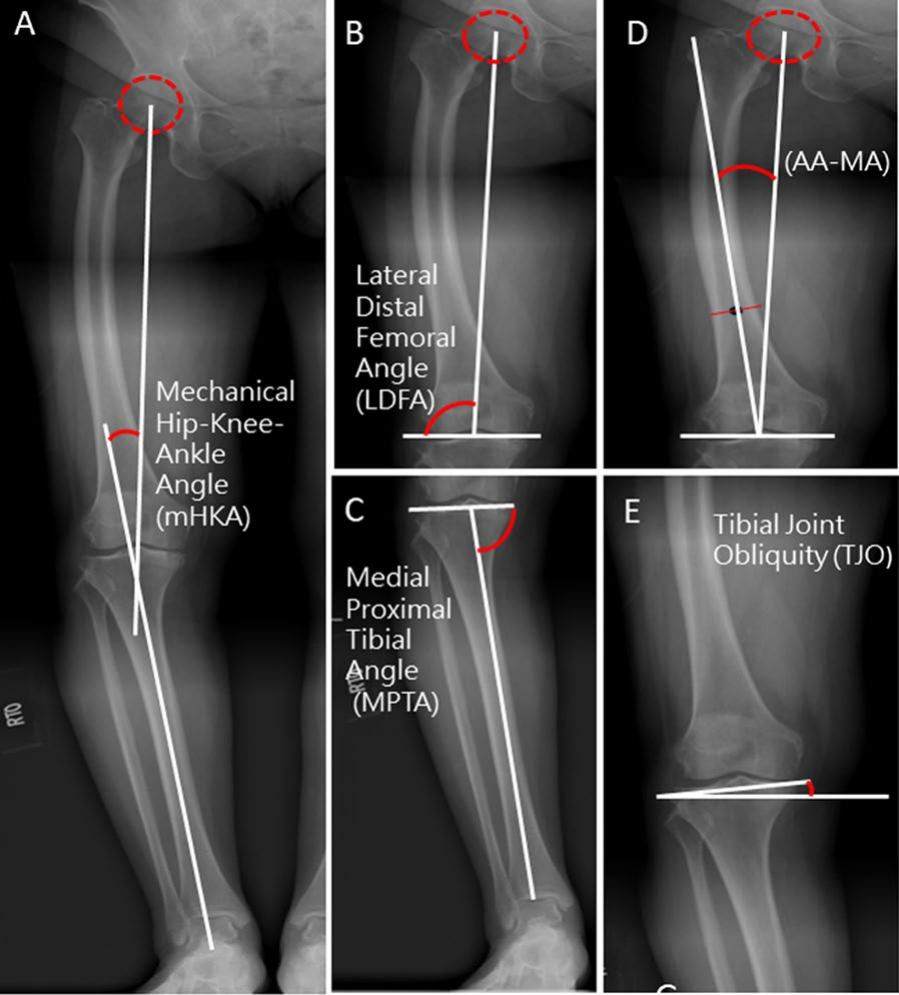
Measurements of key coronal alignment parameters. **A** The mechanical hip–knee–ankle angle (mHKA) is the angle between the femur and tibia’s mechanical axes, with a negative value for varus knee and positive value for valgus alignments. **B** The lateral distal femoral angle (LDFA) is the lateral angle between the femur’s mechanical axis and the distal femur joint line, connecting the lowest points of the femoral condyles. **C** The medial proximal tibial angle (MPTA) is the medial angle between the tibia’s mechanical axis and the proximal tibia joint line, connecting the lowest points of the tibial plateau. **D** The angle between the femoral anatomical axis and mechanical axis (AA-MA). **E** The tibial joint obliquity (TJO) is the angle formed between the floor’s parallel line and the proximal tibia joint line. Positive values represent a lateral open angle, and negative values represent a medial open angle

Coronal radiography of the lower leg and the spine was taken for all patients to identify the spine deformity and previous leg surgery due to fracture or hip arthroplasty. All participants underwent standard digital long-leg radiographs. The mHKA angle was the angle subtended by the mechanical axes of the femur and tibia. The tibial joint line obliquity angle (TJLA), lateral distal femur angle (LDFA), medial proximal tibial angle (MPTA), and angle between the femur anatomic axis and mechanical axis (AA-MA) were measured preoperatively. A detailed description and illustration of the knee alignment angles are shown in Fig. 1.

**Fig. 2:**
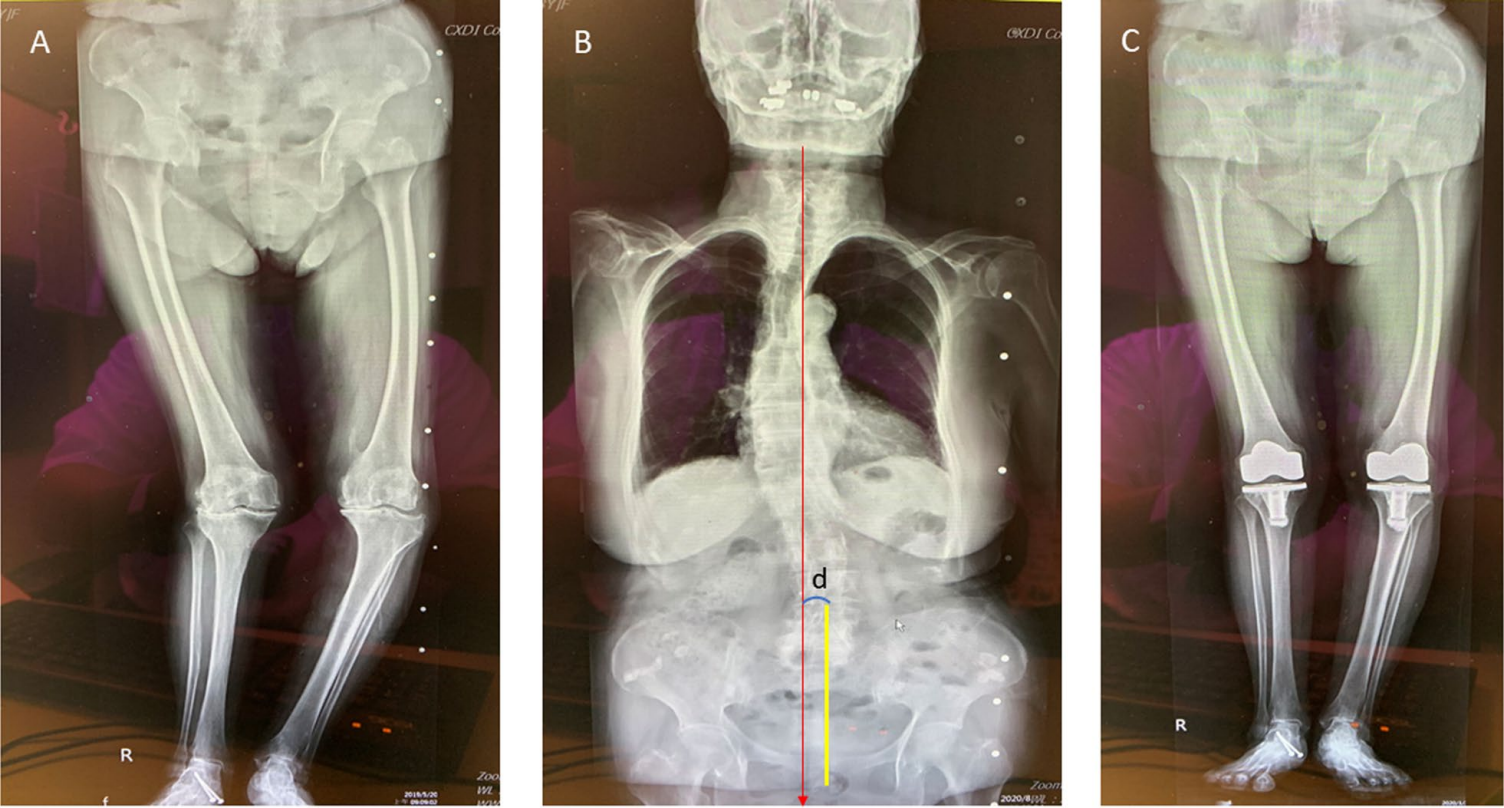
A windswept-deformity patient characterized by a significant degree of malalignment in the lower extremities’ coronal plane, corresponding to a coronal spinal imbalance. **A** The patient’s right valgus knee and left varus knee. **B** The association of windswept deformity with trunk shift, typically toward the valgus knee side, as signified by the deviation of the C7 plumb line (red downward arrow) from the central sacral line (yellow vertical line). “d” represents the deviation distance, with a coronal spinal imbalance being defined as this distance exceeding 2 cm. **C** Substantial alignment enhancement in the lower extremities post the application of the staged, phenotype-considered kinematically aligned total knee arthroplasty procedure

**Fig. 3:**
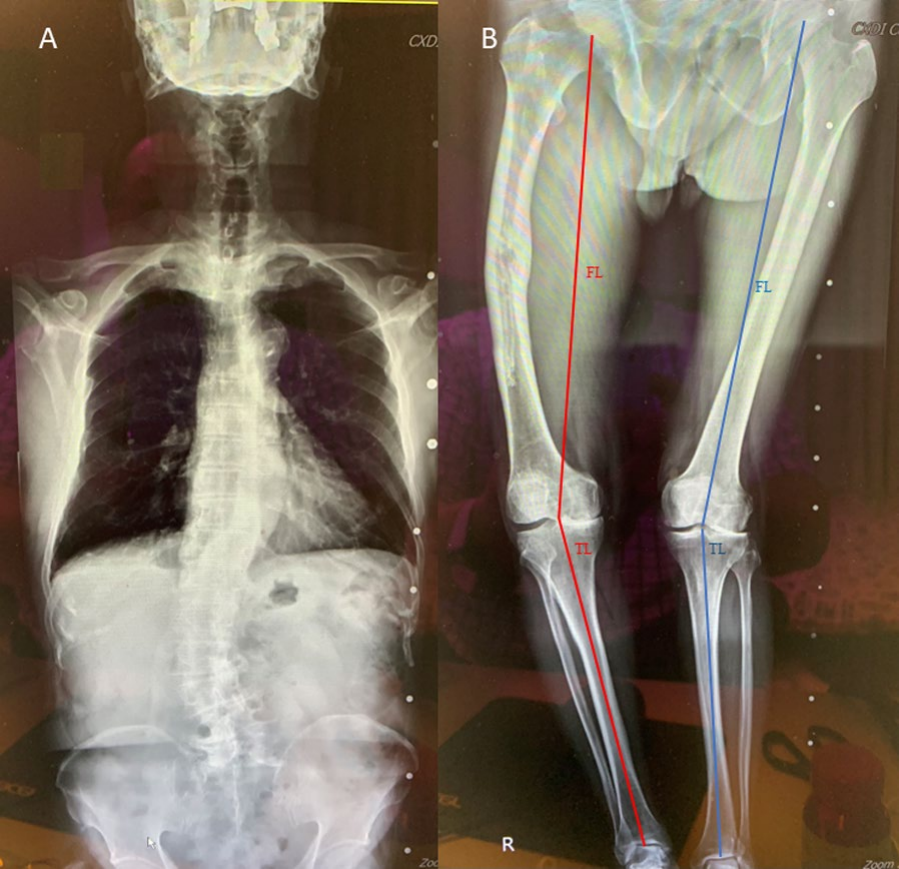
**A**, **B** A windswept-deformity patient having a leg length discrepancy (LLD) caused by an aged femoral shaft fracture. The femur length (FL) is determined by the distance between the femoral head’s center to the knee’s center, while the tibia length (TL) is measured from the knee’s center to the ankle’s center. A leg length discrepancy (LLD) is observed when the sum of the FL and TL varies by more than 1 cm between the varus and valgus sides of the leg

**Fig. 4:**
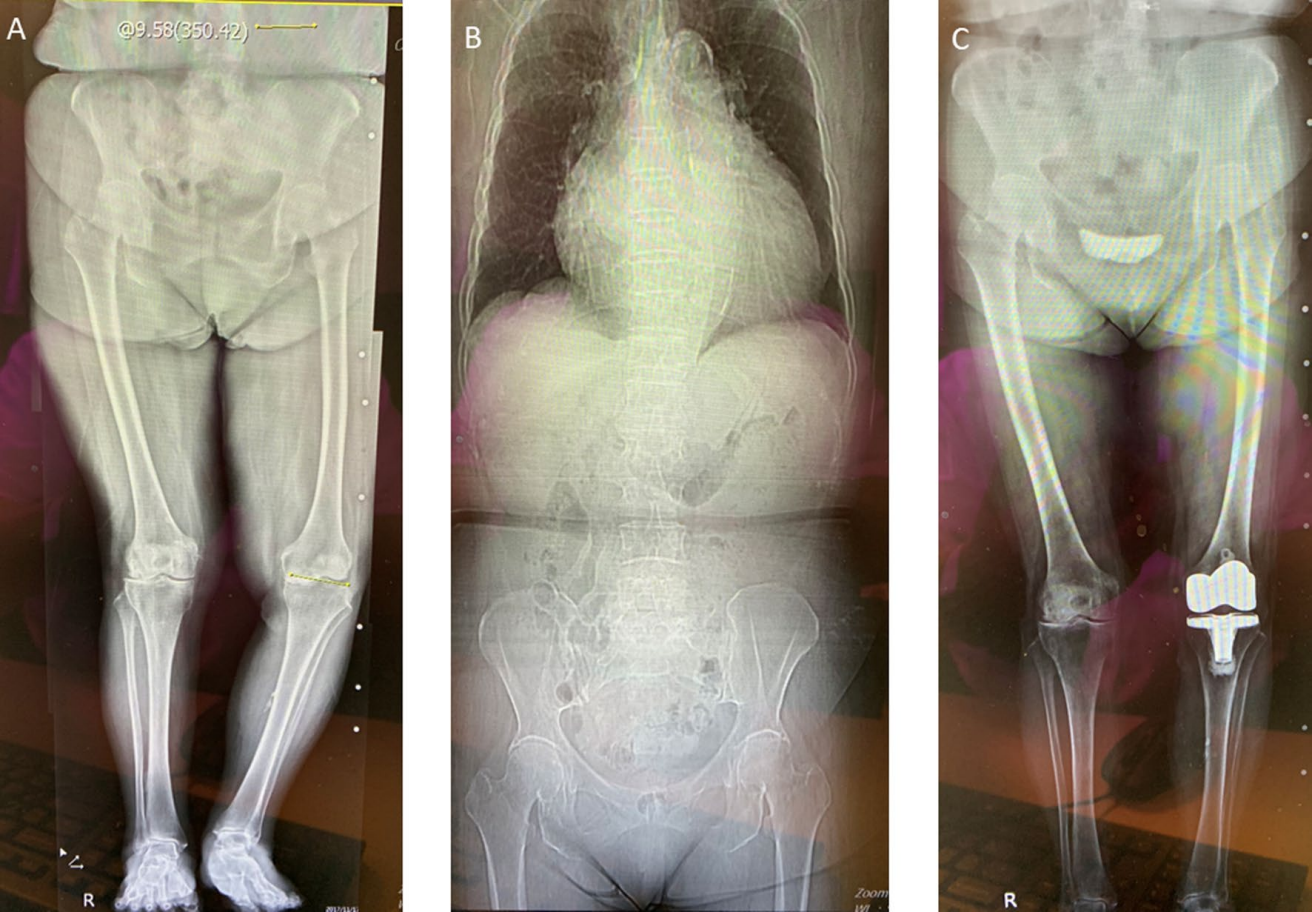
**A**, **B** A windswept-deformity patient lacking a specific condition in spine and leg length. **C** The substantial improvement in malalignment following the application of the staged phenotype-considered kinematically aligned total knee arthroplasty procedure

A full-length coronal plane of the spine was taken to evaluate whether the patient had scoliosis. Long-cassette standing anteroposterior radiographs of the entire spine were obtained preoperatively for all 33 patients. On the coronal films, the coronal balance distance was defined as the horizontal distance between the C7 plumb line (Fig. 2) and the central sacral vertical line. When the distance was more than 2 cm, coronal spine imbalance was defined [17, 18]. The length of the femur (FL) was ascertained by measuring the distance from the center of the femoral head to the center of the knee, while the length of the tibia (TL) was calculated from the center of the knee to the center of the ankle. A discrepancy in leg length (LLD) was noted when the combined measurement of the FL and TL differed by more than 1 cm between the varus and valgus sides of the leg [19]. This discrepancy could be a result of prior fracture or arthroplasty (Fig. 3). If no clear radiographic evidence of spine coronal imbalance or LLD was found, the associated condition of the WSD was classified as unknown (Fig. 4).

Patients were followed up at 3 months, 6 months and 1 year after the operation. Postoperative standing long-leg film was taken to evaluate the postoperative mHKA, LDFA, MPTA, and TJLA. The range of motion (ROM), Oxford Knee Score (OKS), and Knee Society Score (KSS) were evaluated preoperatively and postoperatively at last follow-up. The average follow-up time was 24 months.

### Preoperative planning and original phenotype determination

The classification of knee phenotypes was primarily based on the discrepancy between the mechanical alignment of the LDFA and the MPTA as described below:

The mechanical alignment of the femur was classified into varus, neutral, and valgus. Varus was defined as LDFA ≥ 90°, neutral as 87° ≤ LDFA < 90°, and valgus as LDFA < 87°.

The mechanical alignment of the tibia was characterized as varus, neutral, and valgus. Varus was defined as MPTA < 87°, neutral as 90° ≥ MPTA ≥ 87°, and valgus as MPTA > 90°.

**Fig. 5:**
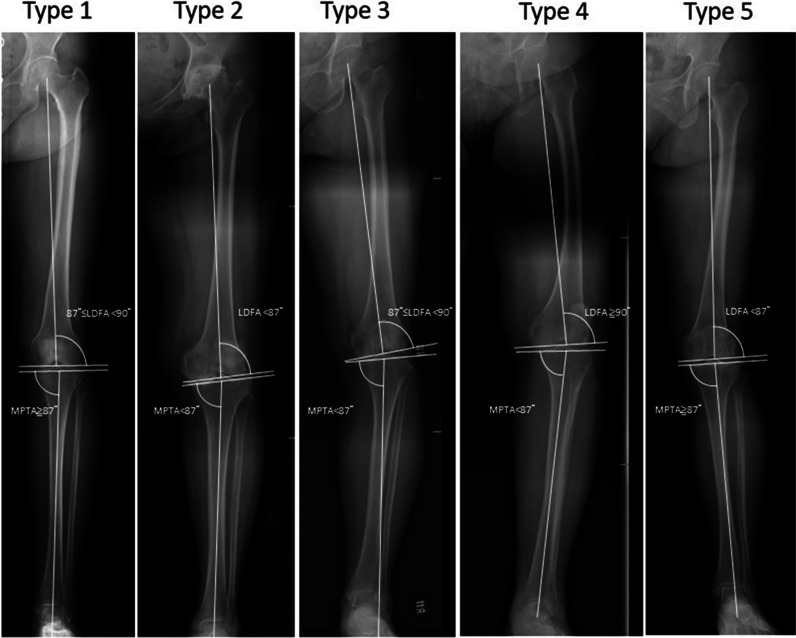
The five most prevalent knee phenotypes categorized based on the various combinations of distal femur and proximal tibia alignment, i.e., the lateral distal femoral angle (LDFA) and medial proximal tibial angle (MPTA)

Based on these varying alignments of the tibia and femur, we can categorize patients into five most common knee phenotypes (Fig. 5), as detailed in our previous study [20]

### Target alignments for each knee phenotype

**Fig. 6:**
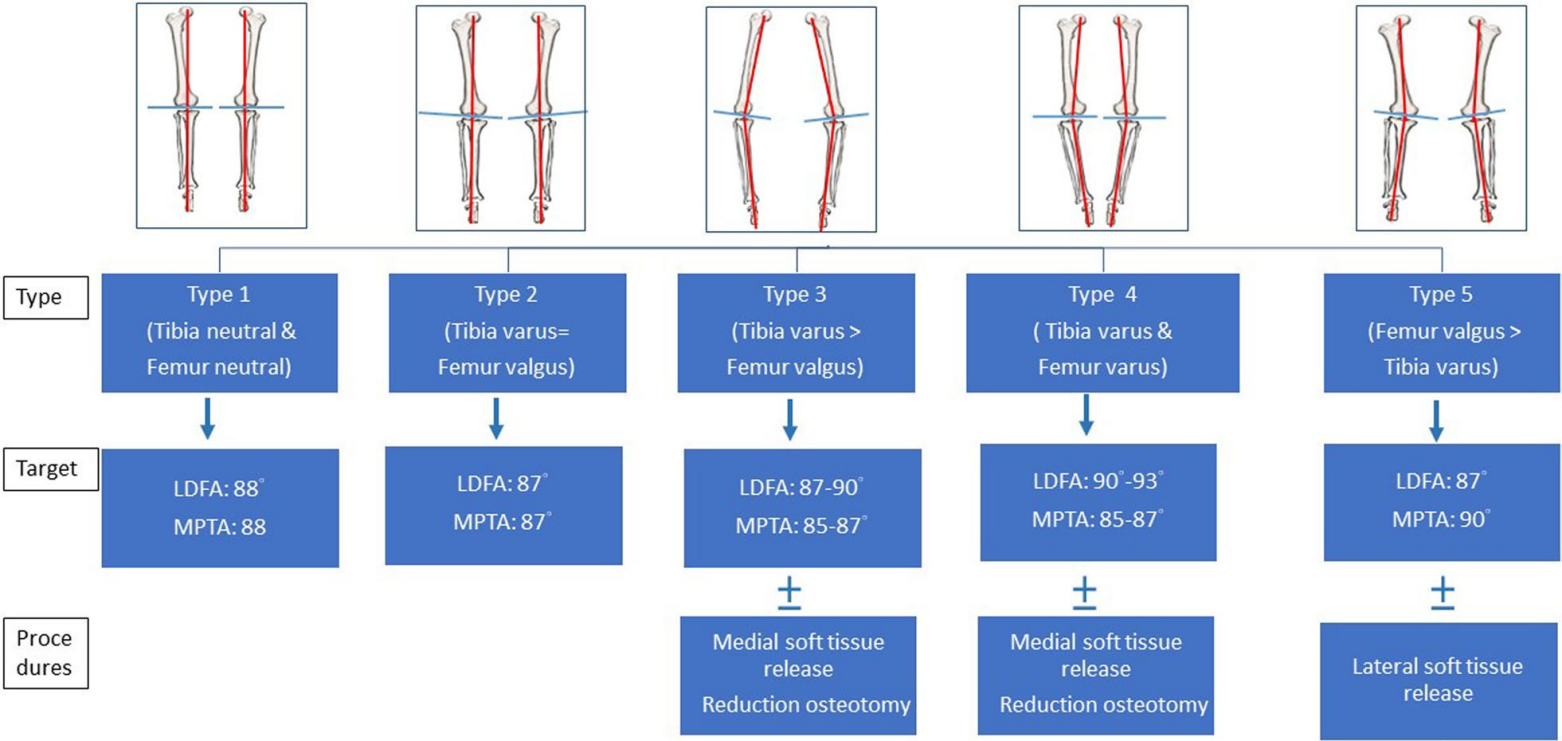
The algorithm indicating the target alignment angles for distal femoral angle (LDFA) and medial proximal tibial angle (MPTA), along with any additional procedures that may be required, according to the five knee phenotypes

Figure 6 illustrates that the need for target alignment and soft tissue procedures can be dictated by the specific characteristics of each knee type. The principles for conducting KA-TKA for each knee type are as follows:

For a type 1 knee, which exhibits a neutral alignment and a transverse joint line, the cuts on the distal femur and proximal tibia are made parallel to the original joint line. The target angles for both the LDFA and MPTA are set at 88°, in accordance with the concept of modified MA-TKA [21].

In the case of a type 2 knee, characterized by a high degree of joint line obliquity, we modified the LDFA and MPTA by 2–3° to reduce this obliquity. The target values for both LDFA and MPTA were set at 87°, aiming to achieve anatomical alignment (AA) and decrease joint line obliquity [22].

For a type 3 knee, marked by a significant degree of tibial varus, the distal femur was cut according to the original joint line, and the target for adjusting the tibial alignment, or MPTA, was set between 85° and 87°.

In the case of a type 4 knee, identified by a simultaneous varus alignment of both tibia and femur and often associated with lateral bowing of the femur and LDFA > 95°, we adjusted the LDFA to the range of 90–93° and the MPTA to 85–87°. This is done to correct the varus alignment of the lower limb. This prevents placing the femoral component into excessive varus alignment, which could potentially increase the rate of knee component loosening [23]. In instances where the varus deformity is particularly severe, it remains necessary to resort to medial soft tissue release and reduction osteotomy techniques to achieve a balanced knee.

For a type 5 knee, characterized by a valgus femur, the designated values were set at 87° for the LDFA and 90° for the MPTA. The LDFA is targeted at 87° because a valgus deformity greater than 3° should be prevented to reduce the risk of patellar instability [24]. Additionally, a lateral release of the iliotibial band and the lateral patellar retinaculum may be necessary to further lower the risk of patellar instability.

We treated the varus side of WSD as a varus knee and the valgus side of the knee as a valgus knee, setting the target for bone cutting according to the phenotype of the knee as done in our previous paper [15]. The rationale behind setting a target alignment for each phenotype was to balance the knee close to its original alignment without excessive soft tissue release. The target was set based on the average angle of each phenotype with mild modification, aiming for the varus knee to remain at 3° of varus, the valgus knee at 3° of valgus, and neutral alignment in a neutral position. For the prevention of patella instability in valgus knees, we routinely performed a lateral release of the iliotibial band and release of the lateral patellar retinaculum if patellar subluxation was noted. We did not perform simultaneous bilateral total knee arthroplasty but, instead, addressed the most painful leg first according to the patient’s description.

### Statistical analysis

Statistical analysis was performed using SPSS (version 25.0; IBM). The Mann–Whitney *U* test was used to compare coronal radiologic parameters between varus and valgus osteoarthritic knees in WSD. The Wilcoxon signed-rank test was used to compare preoperative and postoperative coronal radiologic parameters (mHKA, MPTA, and LDFA) and clinical outcome scores (OKS, CKSS, and ROM). The level of significance was set at *P* < 0.05.

## Results

Out of 1250 patients, a total of 33 patients with windswept deformity (WSD) were identified. The average follow-up time was 24 months, and the prevalence rate during this period was 2.63% (33/1250). The basic characteristics of the 33 patients with WSD are detailed in Table 1. Among these, 10 were male and 23 were female, with an average age of 74.9 years (ranging from 57 to 89 years). Total knee arthroplasties (TKAs) were first performed on the varus leg in 22 patients (67%), but in the valgus leg in 11 patients (33%). Twenty-one out of 33 patients (64%) were associated with coronal spinal imbalance, as determined by a horizontal distance of more than 2 cm between the C7 plumb line (C7PL) and the central sacral vertical line (Fig. 3). Seven patients (21%) were associated with a noticeable LLD greater than 1 cm. Of these, three patients had LLD due to previous lower extremity fractures (Fig. 3), and four patients were affected due to previous hip arthroplasty. The causes for WSD in the remaining five patients were unknown (Fig. 4). In the varus side of the knee joint, 3 patients belong to type 1, 8 to type 2, 13 to type 3, and 9 to type 4. Postoperatively, nine varus knees were corrected to neutral alignment, but the others remained in a varus position more than 3°. In the valgus side of the knee joint, 5 knees belong to type 1, 1 to type 2, and 27 to type 5. Postoperatively, fifteen knees corrected to neutral alignment, but the others remained in a valgus position of more than 3°.

**Table 1:** Basic characteristics of 33 patients with windswept deformity

Variable	Total *n* = 33
Age, years	
Mean ± SD (range)	74.9 ± 9.44 (57 to 89)
Gender	
Male, *n* (%)	10 (30)
Female, *n* (%)	23 (70)
First operated side	
Right, *n* (%)	13 (38)
Left, *n* (%)	20 (62)
Unilateral or bilateral	
Unilateral	26 (79)
Bilateral	7 (21)
Associated condition	
Coronal spinal imbalance, *n* (%)	21 (64)
LLD, *n* (%)	7 (21)
Unknown, *n* (%)	5 (15)
Alignment of first operated leg	
Valgus, *n* (%)	11 (33)
Varus, *n* (%)	22 (67)

*LLD* Leg length discrepancy

The preoperative knee angles of the valgus and varus knees in the 33 WSD patients are detailed in Table 2. Preoperative mHKA and AA-MA were significantly different between the varus and valgus knees (*P* < 0.001 and *P* = 0.022, respectively). There was a significantly decreased LDFA in the valgus knees compared with the varus knees (84.2° versus 88.7°, *P* < 0.001). A significantly decreased MPTA was found in the varus knees compared with the valgus knees (83.6° versus 89.7°, *P* < 0.001). *P* value < 0.05 is defined as statistically significance and expressed as bold form.

**Table 2:** Comparison of preoperative knee angles between the valgus and varus knee in 33 WSD patients

Knee angles	Knee alignment in WSD patient		*P* value
	Valgus (*n* = 33)	Varus (*n* = 33)	
mHKA, °	8.9 ± 4.20 (3.1 to 17.5)	−10.9 ± 6.06 (−23.0 to −3.0)	**< 0.001**
AA-MA, °	5.4 ± 1.52 (1.7 to 8.2)	6.1 ± 1.37 (2.8 to 9.8)	**0.022**
LDFA, °	84.3 ± 2.32 (77.9 to 89.5)	88.8 ± 3.05 (83.0 to 94.2)	**< 0.001**
MPTA, °	89.8 ± 2.18 (86.8 to 95.4)	83.6 ± 2.85 (75.6 to 87.7)	**< 0.001**
TJLA, °	3.0 ± 1.69 (−0.19 to 7.0)	3.8 ± 2.03 (−0.4 to 9.2)	0.088

Values reported as mean ± standard deviation (range). *P* values computed by Wilcoxon signed-rank test. N.C. not compared

Table 3 presents a comparison of preoperative and postoperative knee alignment and clinical outcome scores of 15 operated valgus knees out of the 33 WSD patients. One patient was excluded due to loss to follow-up. Significant differences were observed between preoperative and postoperative mHKA, LDFA, and MPTA in the valgus operated knees. Functional outcomes measured by the ROM, OKS, and KSS improved significantly compared with the preoperative status (*P* < 0.001).

**Table 3:** Comparison of preoperative and postoperative knee alignment in operated valgus knees from WSD patients

Parameter	Preoperative (*n* = 15)	Postoperative (*n* = 15)	*P*-value
mHKA, °	11.4 ± 4.26 (3.6 to 17.5)	2.9 ± 2.05 (−0.2 to 7.2)	**< 0.001**
LDFA, °	83.6 ± 1.37 (81.3 to 85.8)	87.0 ± 1.77 (83.0 to 89.1)	**0.001**
MPTA, °	90.4 ± 2.11 (86.9 to 95.4)	88.5 ± 2.09 (83.0 to 91.3)	**0.047**
ROM	73.3 ± 25.99 (38.0 to 110.0)	120.0 ± 1.93 (40 to 47)	**< 0.001**
Oxford Knee Score	13.0 ± 5.1 (2 to 22)	44.2 ± 1.93 (40 to 47)	**< 0.001**
Knee Society Score	62.0 ± 32.6 (13 to 114)	179.7 ± 9.80 (164to 199)	**< 0.001**

Values reported as mean ± standard deviation (range). *P* values computed by Wilcoxon signed-rank test

Table 4 provides a comparison of preoperative and postoperative knee alignment and clinical outcome scores of 20 operated varus knees from the 33 WSD patients. Four patients were excluded due to loss to follow-up. Significant changes were found in mHKA and MPTA among preoperative and postoperative data. However, no statistically significant change was observed in LDFA between preoperative and postoperative data in the varus operated knees. Functional outcomes measured by ROM, OKS, and KSS showed significant improvement compared with the preoperative status (*P* < 0.001).

**Table 4:** Comparison of preoperative and postoperative knee alignment in operated varus knees from WSD patients

Parameter	Preoperative (*n* = 20)	Postoperative (*n* = 20)	*P* value
mHKA, °	−12.7 ± 6.06 (−4.2 to −23)	−4.6 ± 3.62 (−11.5 to 0)	**< 0.001**
LDFA, °	89.3 ± 3.27 (84.5 to 94.2)	88.9 ± 2.40 (85.7 to 93.8)	0.422
MPTA, °	82.8 ± 3.28 (75.6 to 87.7)	84.9 ± 2.39 (81.5 to 89.8)	**< 0.001**
ROM, °	59.2 ± 23.1 (34.0 to 112.0)	119.0 ± 3.44 (112 to 125)	**< 0.001**
Oxford Knee Score	12.3 ± 6.16 (2 to 26)	42.9 ± 2.79 (38 to 47)	**< 0.001**
Knee Society Score	65.8 ± 24.0 (11 to 98)	176.4 ± 13.94 (148 to 200)	**< 0.001**

Values reported as mean ± standard deviation (range). *P* values computed by Wilcoxon signed-rank test

Table 5 presents a comparison of postoperative knee alignment angles and functional scores between valgus and varus knees in patients with WSD. Significant differences were observed in the knee alignment angles, including mHKA, LDFA, and MPTA between valgus and varus knees (*P* = 0.018). However, no significant statistically differences were observed in functional scores, including ROM, OKS, and KSS between postoperative valgus and varus knees.

**Table 5:** Comparison of postoperative knee alignment angles and function score between valgus and varus knees in patients with WSD. *P* value < 0.05 means statistically significance and expressed as a (*) form

Knee angles	Knee alignments		*P* value
	Valgus (*n* = 15)	Varus (*n* = 20)	
mHKA, °	2.9 ± 2.05 (−0.2 to 7.2)	−4.6 ± 3.62 (−11.5 to 0)	0.018*
LDFA, °	87.0 ± 1.77 (83.0 to 89.1)	88.9 ± 2.40 (85.7 to 93.8)	0.018*
MPTA, °	88.5 ± 2.09 (83.0 to 91.3)	84.9 ± 2.39 (81.5 to 89.8)	0.018*
ROM, °	120.0 ± 1.93 (40 to 47)	119.0 ± 3.44 (112 to 125)	0.109
Oxford Knee Score	44.2 ± 1.93 (40 to 47)	42.9 ± 2.79 (38 to 47)	0.892
Knee Society Score	179.7 ± 9.80 (164 to 199)	176.4 ± 13.94 (148 to 200)	0.285

Values reported as mean ± standard deviation (range).*P values computed by Fisher’s exact test, and others were computed by a Wilcoxon signed-rank test

## Discussion

The two important findings of this study are that (1) the most common associated radiographic findings of WSD were coronal spinal imbalance (64%) and LLD (21%), and (2) phenotype-considered KA-TKA is an effective method for advanced OA knee in WSD patients, offering promising short-term radiologic and functional results.

Another finding of this study is that, among a cohort of 1250 patients, 33 were identified with WSD, resulting in a prevalence rate of 2.64% over an average follow-up period of 24 months. This suggests that WSD is relatively uncommon in the total knee arthroplasty population. For comparison, in Steven Howell’s series [25], only 19 cases were reported out of 2430 patients over 6 years, highlighting a similarly low incidence. However, Howell’s series did not detail any associated deformities in these patients. A systematic review conducted in 2022, examining WSD in total knee arthroplasty patients, found that none of the four articles reviewed reported more than 22 cases [26]. Our study differs from previous ones by utilizing long-leg films instead of the short knee films traditionally used, enabling a more accurate diagnosis of WSD. Additionally, our analysis revealed notable associated conditions related to leg length and coronal spinal balance, offering insights into the management strategies for these patients.

If the patient has WSD, a long spine and leg film is mandatory for the evaluation of coronal malalignment and leg length discrepancy. In our series, more than half (21/33) of WSD patients were associated with coronal spinal imbalance. Scoliosis in childhood is usually associated with lateral pelvic tilting and degenerative scoliosis in adult age, which may cause coronal spinal imbalance and WSD of knee [27]. In such cases of coronal malalignment without leg length discrepancy, the trunk imbalance causes pelvic tilting and leg length discrepancy. Then, to balance the trunk, WSD progressively forms. In WSD patients, if both knee pain and spine stenosis symptoms occur, which one should be addressed first? An interesting paper shows that most spine and arthroplasty surgeons prefer to perform total knee arthroplasty first in patients with windswept deformity, unless the patient has severe neurological problems [28]. In our study, we found that, after the arthroplasty surgery, the trunk shifted from imbalance to more balance. Back pain improved a lot after surgery. The other cause of WSD is due to leg length discrepancy of more than 1 cm due to previous fracture or arthroplasty. Usually, leg length discrepancy is the major cause of trunk imbalance, and the other leg deformity opposite to the original knee.

Existing literature rarely covers surgical strategies for treating patients with windswept deformity (WSD). The treatment approach for osteoarthritis (OA) in a knee affected by WSD can vary significantly between the legs. In such a scenario, it is imperative to use an intraoperative caliper to ascertain the bone resection thickness at every critical surgical phase before moving on to the subsequent stage. This approach enables the surgeon to make necessary alignment adjustments during the KA-TKA process. A methodology adopted by Howell et al., which involves the use of calipered KA-TKA with a cruciate retaining knee, has shown effectiveness in treating both varus and valgus knees in WSD patients [25]. Through this method, the postoperative alignment difference in terms of lateral distal femoral angle (LDFA), medial proximal tibial angle (MPTA), and mechanical hip–knee–ankle angle (mHKA) between the paired knees with varus and valgus deformities can be corrected to 1° or less. This approach yielded similar postoperative MPTA in varus and valgus knees. The postoperative Oxford Knee Score (OKS) and Forgotten Joint Score (FJS) were reported to be excellent, at 47 and 90 points, respectively. Moreover, no statistical difference was observed in OKS and FJS between the valgus and varus knees [25]. In our approach, we treated valgus and varus knees based on the distinct alignment targets for different knee types. We observed significant differences in postoperative medial proximal tibial angle (MPTA) between valgus and varus knees, measuring 88.5° and 84.9°, respectively. Moreover, a residual valgus of 2.9° and varus of 4.6° were found in valgus and varus knees in our study, respectively. We approached each varus and valgus knee independently, adjusting our treatment according to the targeted bone cut thickness, MPTA, and lateral distal femoral angle (LDFA) for each knee type, as detailed in our previous publication [15, 16]. Our decision to use target LDFA and MPTA angles as guides is driven by the understanding that pre-arthritic angles may alter due to arthritic bone wear. We employ soft tissue release and reduction osteotomy techniques to achieve as close to neutral alignment as possible, acknowledging the impact of mechanical alignment on prosthesis longevity. In most cases, we adjust the alignment of the valgus knee close to neutral. However, the varus knee typically retains a slight varus alignment after correction. We do not aim for complete correction to mechanical neutrality, given that WSD is often associated with trunk imbalance due to scoliosis or leg length discrepancy (LLD). Overcorrection could lead to a disrupted balance of the trunk, back pain, altered knee joint line, and an increased tibial adduction moment [29–34]. Impressive postoperative clinical outcomes and high patient satisfaction levels observed in our 2-year follow-up suggest that our approach is effective in treating WSD patients with advanced OA knees. Throughout the follow-up period, patients reported no major issues such as back pain or leg length discrepancies post-surgery. When managing WSD, it is particularly crucial to tailor the correction angle for valgus deformities to each individual patient’s needs [4]. In our study, we provided a comprehensive method for bone resection according to the different knee phenotypes [15, 16, 20].

Another issue is whether WSD with advanced OA knee is a good indication for simultaneous bilateral TKA (SBTKA). Though SBTKA has been reported to have an increased risk for all complications even in the healthiest patients [35], some authors considered it to be advantageous in comparing the limb alignment, length, and use of autologous bone from bony resections to build bony defects during the surgery while both legs are sterile-draped [36, 37]. In our study, we suggested that patients receive staged TKA on the most painful leg first. The majority (22/33) of the patients chose the varus leg first. Symptoms of the other leg were usually greatly improved due to the change in lower limb alignment. Only seven patients chose to receive the second leg TKA in the 2-year follow-up period. Because we did not do simultaneous bilateral TKA, after surgery, we will use foot padding block test to check leg length discrepancy. If any leg length discrepancy is noted, we will add padding to the shoe to balance the lower extremity to prevent back pain after surgery.

This study has several limitations. First, the 2-year follow-up time is relatively short to assess long-term complications such as aseptic loosening, which may be affected by component alignment [38, 39], although positive 10-year results of KA-TKA have been published [40]. The long-term survivorship of varus tibial component may be due to the ground-parallel joint line. Second, these results correspond to a small number of patients (*n* = 33) and should be confirmed by future studies with larger sample size. Third, the present study used only anteroposterior radiographs, without a lateral view, for the radiological evaluation. Thus, we only examined the coronal alignment. Lastly, because of the retrospective nature of our study, we lacked a control group to evaluate surgical outcomes. Future research should conduct comparative studies on different approaches to assess the effectiveness of surgical strategies.

## Conclusions

A high percentage of patients with WSD exhibited coronal spinal imbalance and leg length discrepancy. Phenotype-considered KA-TKA effectively provided alignment targets for the treatment of both varus and valgus knees in patients with WSD, achieving excellent short-term outcomes and acceptable knee alignment.

## Data Availability

The data that support the findings of this study are available from the corresponding author upon reasonable request.
